# *N*-Acetylcysteine inhalation improves pulmonary function in patients received liver transplantation

**DOI:** 10.1042/BSR20180858

**Published:** 2018-09-28

**Authors:** Xiaoyun Li, Xiaoxia Wei, Chaojin Chen, Zheng Zhang, Dezhao Liu, Ziqing Hei, Weifeng Yao

**Affiliations:** 1 Department of Anesthesiology, The Third Affiliated Hospital of Sun Yat-sen University, Guangzhou City, People’s Republic of China; 2 Department of Anesthesiology, The People’s Hospital of Guangxi Zhuang Autonomous Region, Nanning City, People’s Republic of China

**Keywords:** acute lung injury, liver transplantation, model for end-stage liver disease, mucolytic, randomized trial

## Abstract

Postoperative pulmonary complications (PPCs) following orthotopic liver transplantation (OLT) are associated with high morbidity and mortality rates. The effect of *N*-acetylcysteine (NAC) inhalation on the incidence of PPCs and the outcomes of patients undergoing OLT is unknown. This prospective randomized controlled clinical trial was conducted to investigate the effect of NAC inhalation during OLT on PPCs. Sixty patients were randomly assigned to the NAC group (*n* = 30) or the control group (*n* = 30) to receive inhaled NAC or sterilized water, respectively, for 30 min before surgery and 3 h after reperfusion. The incidence of early PPCs and outcomes including survival rate were assessed. Biomarkers including tumor necrosis factor (TNF)-α, interleukin (IL)-8, Clara cell secretory protein (CC16), intercellular adhesion molecule (ICAM)-1, and superoxide dismutase (SOD) were measured in exhaled breath condensate (EBC) at T1 (before surgery) and T2 (at the end of operation) as well as in serum at T1, T2, T3 (12 h after operation), and T4 (24 h after operation). A total of 42 patients (20 in the NAC group and 22 in the control group) were enrolled in the final analysis. Atomization inhaled NAC significantly reduced the incidence of PPCs after OLT. The levels of TNF-α, IL-8, CC16, and ICAM-1 in EBC were significantly lower, and SOD activity was higher, at T2 in the NAC group; similar data were found in serum at T2, T3, and T4. In summary, perioperative NAC inhalation may reduce the incidence of PPCs and improve patient outcomes after OLT.

## Introduction

Orthotopic liver transplantation (OLT) is one of the most effective treatments for end-stage liver disease and acute liver failure. However, postoperative pulmonary complications (PPCs) following OLT are associated with high morbidity and mortality rates. The incidence of PPCs, including pleural effusion, atelectasis, pneumonia, acute lung injury (ALI), and acute respiratory distress syndrome (ARDS), is higher than 80%, with a wide variation across reports depending on the definition adopted [1,2]. PPCs are associated with long-term mechanical ventilation, long hospital stays, and poor outcomes [3,4]. The origin of PPCs is multifactorial. In our previous study, upon multivariate regression analysis, preoperative model for end-stage liver disease (MELD) score >19 was found to be an independent risk factor for PPCs, and high preoperative albumin level was found to be a protective factor against PPCs diagnosed on the basis of the Melbourne Group Scale Version 2 (MGS-2) [5]. Prevention of infectious risks, early diagnosis, and treatment are key to improve survival of patients undergoing OLT [6–9]. However, effective preoperative and intraoperative preventive measures are lacking.

*N*-Acetylcysteine (NAC), the precursor of l-cysteine and reduced glutathione (GSH), has been widely used as a mucolytic agent in several lung disorders, where it improves small airway function. NAC acts as a free oxygen radical scavenger [10,11] and has anti-inflammatory activity both *in vitro* and *in vivo* [12]. Furthermore, NAC attenuates liver ischemia–reperfusion (I/R) [13,14] and protects the lungs against hepatic I/R damage in the studies of Weinbroum et al. [15,16]*.* NAC can be administered orally, intravenously, or via aerosol. In our previous animals study, we also found NAC intraperitoneal injection could attenuate lung injury after liver transplantation [17]. However, inhalation therapy has the advantage of lower dosage, more rapid onset, and less side effects because of the direct effects on the airways. Thus, inhalation therapy has been used for the treatment of asthmantia, chronic obstructive pulmonary disease (COPD), ALI, and other pulmonary diseases [18].

We hypothesized that inhaled NAC would decrease the incidence of PPCs after OLT and improve patient outcomes. This clinical trial was conducted to investigate the effect of preoperative and intraoperative NAC inhalation during OLT on PPCs.

## Methods

### Study design

This prospective, randomized, single-blinded, single-center study was conducted in a university-based teaching hospital. The study was performed in accordance with the Declaration of Helsinki and approved by the Institutional Review Board of The Third Affiliated Hospital of Sun Yat-sen University (No. [2014] 2-93). The study was registered by Xiaoxia Wei before patient enrollment in the Chinese clinical trial registry center on October 19, 2014 (ChiCTR-TRC-14005169). Written informed consent was obtained from all patients.

### Study patients and protocol

The present study was performed at The Third Affiliated Hospital of Sun Yat-sen University. All 60 consecutive patients undergoing OLT from April 2014 to April 2016 were assessed for inclusion in the study. The follow-up was 12 months. Inclusion criteria were: (1) patients aged between 18 and 65 years old; (2) patients who signed an informed consent form; (3) patients with no other severe organ dysfunction; and (4) organs procured from donors. Exclusion criteria were: (1) re-transplantation; (2) living donor transplantation; (3) combined liver–kidney transplantation; (4) patients with other serious diseases; and (5) patients with moderate to severe preoperative respiratory test findings. If a patient experienced massive blood loss (more than 10000 ml), cardiac arrest, or acute heart failure during the surgery, the study was stopped and the patient was excluded from the final analysis. Other exclusion criteria included death within 24 h after operation and loss to follow-up.

All donated organs were obtained from the Chinese Organ Procurement Organization and allocated to patients by the China Organ Transplant Response System as per the Chinese human organ-transplant regulation. We confirm that no donor organs were derived from incarcerated persons, that unwilling organ donations were never performed, and that no organs were procured from persons whose death was due to capital punishment. All transplant patients (donors and recipients) were reviewed by Human Organ Transplant Technology Clinical Application Committee and the Ethics Committee of the Third Affiliated Hospital of Sun Yat-sen University using strict criteria for acceptance.

Patients were randomly divided into two groups: (1) the control group, which received atomization inhalation of 3 ml sterile water for 30 min before surgery and 3 h after reperfusion; and (2) the NAC group, which received atomization inhalation of 3 ml NAC (10%) (Fluimucil, Zambon Group, Italia; No. H20110405) for 30 min before surgery and 3 h after reperfusion. Two researchers (Z.H. and G.L.) enrolled the participants. The study investigators (X.L. and X.W.) who collected the data and assessed the outcomes were unaware of the allocation.

The following baseline parameters were collected before surgery: age, weight, gender, body mass index (BMI), MELD scores, preoperative lung disease status, preoperative smoking history, preoperative respiratory test, serum albumin, serum creatinine, and international normalized ratio (INR). The following intraoperative clinical variables were recorded, which might be related to PPCs [1]: cold ischemia time, anhepatic time, operation time, infusion volume (red blood cells [RBCs], plasma, cryoprecipitation, and crystalloid solution), and loss (blood loss and urine volume).

### Sample size

Our primary outcome measure was the incidence of early PPCs. A pilot study in our group found that the incidence of early PPCs was 80% and 50% for the control group and the NAC group, respectively. To achieve acceptable results, the present study required a sample size of 20 per group with a power of 90% and a type I error of 5%. In order to account for any dropouts, 30 patients were recruited to each study group.

### Anesthesia and surgical procedure and monitoring

All surgical procedures were performed under general anesthesia. Anesthesia was induced with intravenous midazolam (0.1 mg/kg), sufentanil (0.2–0.5 μg/kg), propofol (1–1.5 mg/kg), and cisatracurium (0.2 mg/kg), and maintained with end-tidal sevoflurane (2–2.5%). Respiratory parameters were adjusted to maintain end-tidal CO_2_ (ETCO_2_) at 35–45 mmHg. Continuous direct arterial pressure was monitored via the left radial artery, and a pulmonary artery catheter was then inserted to measure cardiac output. Any hypotension was treated with fluids and vasoactive agent infusion (e.g., norepinephrine and dopamine). The body temperature was kept at 36–37°C by convection warming.

The standard approach for liver transplantation was a bilateral subcostal incision with a midline extension. OLT was performed using a classic technique as described previously [19]. No venovenous bypass was performed. OLT was divided into pre-anhepatic (from the beginning of the operation to vascular clamping), anhepatic (from vascular clamping to reperfusion of the portal vein and inferior vena cava), and neohepatic phase (from reperfusion of donor liver to the end of operation). Any adverse reactions to NAC were treated and recorded. After surgery, all recipients were transferred to the intensive care unit (ICU), where they received uniform standard postoperative treatments.

### Exhaled breath condensate and serum sample collection and biomarker detection

Biomarker levels in the exhaled breath condensate (EBC) can reflect pulmonary disease status. We used special glass equipment, which was devised by our team and has been described elsewhere [20], to collect EBC non-invasively for approximately 30 min at T1 (before surgery) and T2 (at the end of operation). Venous blood samples were collected at T1, T2, T3 (12 h after operation), and T4 (24 h after operation). Blood samples were centrifuged for 5 min at 3000 rpm after standing for 2 h at room temperature. The serum was collected. The EBC and serum were stored at 80°C until further use.

Enzyme-linked immunosorbent assay kits were used to measure the concentration of tumor necrosis factor (TNF)-α, interleukin (IL)-8, surfactant protein-D (SP-D), Clara cell secretory protein (CC16), and intercellular adhesion molecule-1 (ICAM-1) both in EBC and serum. The xanthine oxidase method kit was used to measure the activity of superoxide dismutase (SOD) both in EBC and serum. All kits were provided by Wuhan USCN Business Co. Ltd, Wuhan.

### Outcome measures

The primary outcome measure was the incidence of early PPCs (within 1 month after OLT). The presence of PPCs was screened according to MGS-2 [21–25] (Supplementary Table S1). PPCs were diagnosed if four or more variables were present.

**Table 1:** Perioperative baseline demographics

Variables	Control group (*n* = 22)	NAC group (*n* = 20)	*P* value
**Preoperative variables**			
Age (years)	48.2 ± 9.8	43.5 ± 14.3	0.218
Weight (kg)	60.2 ± 10.3	58.5 ± 12.5	0.632
Body mass index (kg/m^2^)	21.4 ± 3.8	20.1 ± 5.5	0.375
Men/women	19/3	18/2	0.716
MELD ≥ 19 points, *n* (%)	9 (41)	7 (35)	0.694
Preoperative lung disease, *n* (%)	11 (50)	9 (45)	0.746
Albumin (g/l)	37.4 ± 6.7	35.2 ± 5.6	0.130
Hemoglobin (g/l)	107.3 ± 28.5	99.5 ± 23.3	0.342
Smoking history, *n* (%)	5 (23)	7 (35)	0.379
Indication for liver transplantation			
Severe hepatitis, *n*	8	8	0.809
Decompensated cirrhosis, *n*	6	5	0.867
Hepatocellular carcinoma with or without cirrhosis, *n*	8	7	0.927
Ascitis, *n*	6	6	0.845
International normalized ratio	2.3 ± 0.9	2.0 ± 1.0	0.494
Serum creatinine (μmol/l)	84.5 ± 42.0	106.7 ± 65.7	0.194
**Intraoperative variables**			
Cold ischemia time (h)	5.93 ± 0.68	6.12 ± 0.43	0.281
Anhepatic time (min)	43.65 ± 6.97	45.58 ± 5.43	0.163
Operation time (h)	6.83 ± 0.78	7.29 ± 0.97	0.097
RBC transfusion unit (U)	20.8 ± 16.4	20.7 ± 17.5	0.989
Fresh frozen plasma (ml)	2268 ± 1073	2105 ± 931	0.601
Cryoprecipitate (ml)	985 ± 360	1150 ± 478	0.211
Urine output (ml)	865 ± 387	879 ± 332	0.125

Abbreviations: MELD, model for end-stage liver disease; NAC, *N*-acetylcysteine; RBC, red blood cell.

A broader definition of PPCs includes the incidence of ALI, ARDS, atelectasis, pneumothorax, pleural effusion, and pneumonia (Supplementary Table S2). All complications were diagnosed by two independent doctors (X.L. and X.W.).

**Table 2:** Incidence of pulmonary complications after orthotopic liver transplantation

Complications	Control group (*n* = 22)	NAC group (*n* = 20)	*P* value
PPC, *n* (%)	16 (72.3)	8 (40)	0.032
ALI, *n* (%)	8 (36.4)	2 (10)	0.045
ARDS, *n* (%)	6 (27.3)	1 (5)	0.053
Atelectasis, *n* (%)	0 (0)	2 (10)	0.129
Pneumothorax, *n* (%)	2 (9)	2 (10)	0.920
Pleural effusion, *n* (%)	20 (90)	19 (95)	0.607
Pneumonia, *n* (%)	18 (82)	12 (60)	0.118
Fungal infection, *n* (%)	6 (27)	1 (5)	0.053
Bacterial infection, *n* (%)	2 (9)	8 (40)	0.019
Combined infection, *n* (%)	5 (23)	2 (10)	0.269
Unknown infection, *n* (%)	5 (23)	1 (5)	0.101

Abbreviations: ALI, acute lung injury; ARDS, acute respiratory distress syndrome; NAC, *N*-acetylcysteine; PPC, postoperative pulmonary complication.

Secondary outcome measures included the incidence of suction with the fiber bronchoscope, thoracentesis, re-intubation, mechanical ventilation duration, ICU and hospitalization stay, hospitalization expenses, 30-day and 12-month survival rates, and the death reasons at 12 months whether related to pulmonary disease. The interval time between the OLT procedure and OLT-related death or the end of the 12-month follow-up was defined as the survival time. The expression level of TNF-α, IL-8, SP-D, CC16, ICAM-1, and SOD in EBC at T1 and T2 and in serum at T1, T2, T3, and T4 was also measured.

### Statistical analysis

Statistical analyses were performed using SPSS 22.0 software (SPSS Inc., Chicago, IL). One-sample Kolmogorov–Smirnov test was used to test the normality of quantitative data. Quantitative variables of normal distribution were presented as mean ± standard deviation (SD). One-way analysis of variance was used to compare the differences between groups, and the paired *t*-test was used to compare the differences within groups. Non-normally distributed quantitative variables were presented as median (interquartile range [IQR]). The Wilcoxon rank sum test was used to compare the differences between groups. Qualitative data were presented as percentage/composition ratio; the Pearson’s chi-square test or Fisher’s exact probabilities were used to compare the differences between groups. Survival analysis was performed to compare the 30-day and 12-month survival rates by the Kaplan–Meier curve. Differences were considered significant when the two-tailed *P* values were <0.05.

## Results

**Figure 1:**
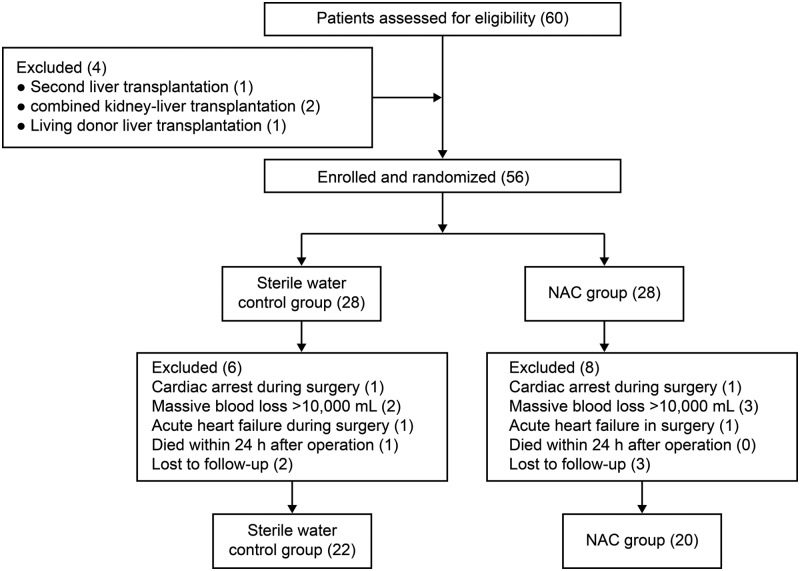
Flow chart of patient selection

A total of 60 consecutive patients scheduled for OLT were randomly assigned to the control group (*n* = 30) or the NAC group (*n* = 30). After excluding patients who met the exclusion criteria or were lost to follow-up, a total of 42 patients were included in the final analysis (22 in the control group and 20 in the NAC group). A flow chart of patient selection is shown in Figure 1. The overall preoperative and operative characteristics of patients in the two groups were well matched (Table 1). There was no significant difference between the two groups with regard to loss to follow-up.

### Primary outcome measures

The main methods for diagnosis of PPCs include MGS-2 and the broad definition of PPCs. Based on the broad definition of PPCs, the incidences of atelectasis, pneumothorax, pleural effusion, ALI, ARDS, and pneumonia were similar between the two groups (all *P* > 0.05) (Table 2). However, based on the MGS-2 score, the control group had a significantly higher PPC rate compared with the NAC group (73% vs. 40%, *P* = 0.032) (Table 2).

Of the 42 patients, 30 (71%) developed at least one respiratory infection episode (including pneumonia) within 1 month postoperatively. Isolated bacterial infection was seen in only 2 (11%) of 18 patients in the control group and in 8 (67%) of 12 patients in the NAC group (*P* = 0.019). Fungal, combined, or unknown infection comprised the major reasons of pneumonia in the control group, but not in the NAC group (27% vs. 5%, *P* = 0.053; 23% vs. 10%, *P* = 0.269; and 23% vs. 5%, *P* = 0.101, respectively) (Table 2).

Most patients (18/22) developed and combined more than three kinds of coexisting pulmonary complications within 1 month postoperatively in the control group, a rate that was higher than that of the NAC group (82% vs. 50%, *P* = 0.028).

### Secondary outcome measures

Compared with the control group, the NAC group had a significantly shorter ICU (6.3 ± 4.5 days vs. 3.9 ± 1.8 days, *P* = 0.026) and hospitalization stay (58.0 ± 24.4 days vs. 43.0 ± 17.6 days, *P* = 0.029) accompanied by significantly lower hospitalization costs (41.0 ± 14.1 vs. 31.4 ± 11.4 × 10000 yuan, *P* = 0.020). There were no statistically significant differences between the two groups with respect to suction with fiber bronchoscope, thoracentesis, re-intubation, and mechanical ventilation duration (Table 3).

**Table 3:** Outcomes of the two groups patients

Outcome	Control group (*n* = 22)	NAC group (*n* = 20)	*P* value
Bronchoscopy sputum suction, *n*	6	5	0.867
Thoracentesis, *n*	5	3	0.524
Reintubation or tracheotomy, *n*	6	2	0.155
Duration of mechanical ventilation (h)	23.5 (12–233)	17.5 (4–87.5)	0.154
ICU stay, days	6.3 ± 4.5	3.9 ± 1.8	0.026
Hospitalization stay, days	58.0 ± 24.4	43.0 ± 17.6	0.029
Cost, 10 thousand yuans	41.0 ± 14.1	31.4 ± 11.4	0.020
30-day survival, *n* (%)	19 (82)	19 (95)	0.341
12-month survival, *n* (%)	15 (68)	18 (90)	0.085
12-month mortality related to lung disease, *n* (%)	6 (27)	0 (0)	0.012

Abbreviations: ICU, intensive care unit; NAC, *N*-acetylcysteine.

**Figure 2:**
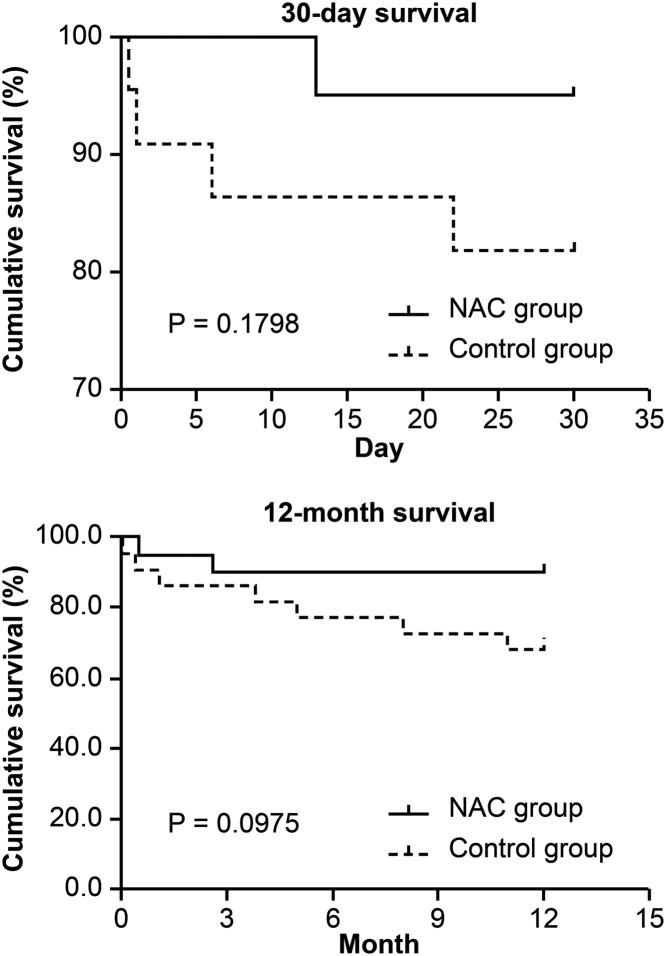
30-day and 12-month survival of the patients included in the present study

Kaplan–Meier curves revealed a trend toward higher 30-day and 12-month survival rates in the NAC group compared with the control group, yet with no significant differences between the two groups, Notably, the 12-month mortality rate related to pulmonary disease was significantly lower in the NAC group than in the control group (*P* = 0.012) (Figure 2, Table 3).

### Inflammatory markers

**Figure 3:**
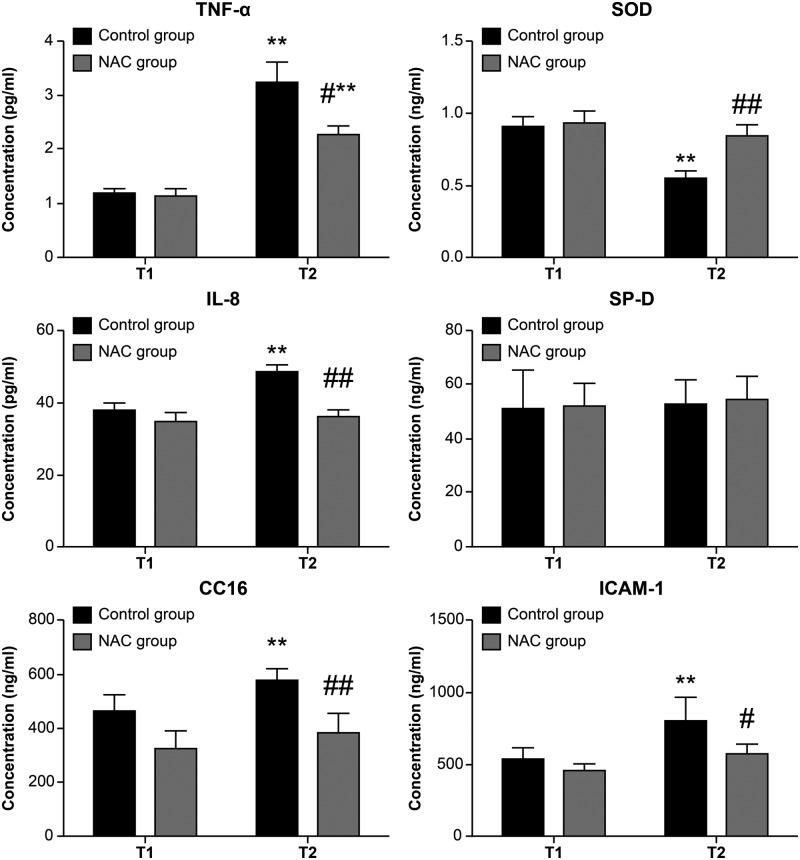
The expression levels of TNF-α, IL-8, CC16, and ICAM-1 in EBC of the two groups *n* = 22 in the control group and *n* = 20 in the NAC group, data were showed as mean ± SD, **P < 0.01 vs. T1 control group, ^#^*P* < 0.05 vs. T2 NAC group, ^##^*P* < 0.01 vs. T2 NAC group.

The expression levels of TNF-α, IL-8, CC16, and ICAM-1 were significantly increased, and SOD activity was significantly decreased, at T2 in EBC of the NAC group as compared with T1 in the control group (*P* < 0.05 or *P* < 0.01), while only TNF-α was significantly increased from T1 to T2 in the NAC group (*P* < 0.01 or *P* < 0.05). Compared with the control group, the expression levels of TNF-α, IL-8, CC16, and ICAM-1 were significantly lower, and SOD activity was higher, at T2 in the NAC group (*P* < 0.05 or *P* < 0.01). No significant differences were observed in SP-D expression (Figure 3).

**Figure 4:**
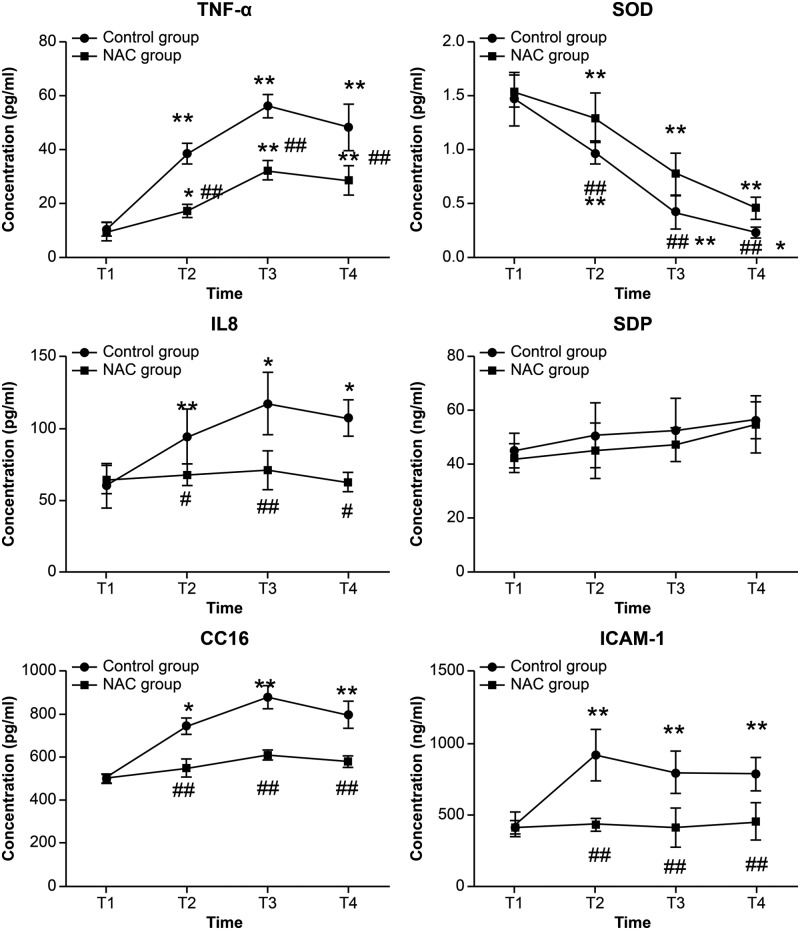
The expression levels of TNF-α, IL-8, CC16, and ICAM-1 in serum of the two groups *n* = 22 in the control group and *n* = 20 in the NAC group, data were shown as mean ± SD, *P < 0.05 vs. T1 control group, **P < 0.01 vs. T1 control group, ^#^*P* < 0.05 vs. the same time point control group, ^##^*P* < 0.01 vs. the same time point control group.

Similarly, the serum levels of TNF-α, IL-8, CC16, and ICAM-1 were significantly increased, and SOD activity decreased, at T2, T3, and T4 in the control group, while only the level of TNF-α significantly increased, and SOD activity decreased, at T2, T3, and T4 (*P* < 0.05, *P* < 0.01). The serum levels of TNF-α, IL-8, CC16, and ICAM-1 were significantly lower, and SOD activity was higher, at T2, T3, and T4 in the NAC group as compared with the control group (*P* < 0.05 or *P* < 0.01) (Figure 4).

### Side effects

No side effect related to NAC inhalation was observed.

## Discussion

The incidence of PPCs after liver transplantation is high and is associated with high mortality rates [1,26,27]. In the present study, we applied both MGS-2 and the broad definition of PPCs to evaluate the effect of inhaled NAC in patients who had undergone OLT. Our results showed that NAC inhalation had no significant effect on PPCs according to the broad definition. Surprisingly, NAC inhalation was related to a lower incidence of PPCs as defined according to MGS-2. Perioperative NAC inhalation was also associated with better early prognoses, including shorter ICU and hospital stay and accompanying lower hospitalization costs. While our data showed that NAC inhalation could not seemingly influence the survival rate at 30 days and 12 months, the percentage of patients whose reason of death was related to pulmonary disease was relatively lower in the NAC group.

Any conditions affecting the respiratory tract that would adversely influence the clinical course of a patient after surgery could be defined as PPCs, which serves the broad definition of PPCs [28]. According to these criteria, PPCs include respiratory failure, respiratory infection including pneumonia, atelectasis, pneumothorax, pleural effusion, ALI, ARDS, and various forms of upper airway obstruction [28]. Accordingly, the incidences of PPCs vary broadly across different reports from less than 10% to more than 80% depending on the criteria adopted, which hinders the early detection and treatment of PPCs [28,29]. According to the broad definition of PPCs, in the present study, NAC inhalation did not reduce the incidence of PPCs. However, further analysis revealed that 89% and 88% patients in the control group and 22% and 50% patients in the NAC group sustained refractory pulmonary infections and combined more than three types of PPCs according to the broad definition, suggesting that NAC inhalation might reduce the incidence of PPCs.

MGS-2 uses eight dichotomous factors to score PPCs, considering not only objective symptoms and evidences (including temperature >38°C, white cell count >11.2, use of respiratory antibiotics, diagnosis of pneumonia, chest X-ray report of atelectasis/consolidation, purulent sputum, and SpO_2_ <90% on room air) but also subsequent treatment for respiratory problems such as longer ICU stay and re-admission. Therefore, the MGS-2 relates the diagnoses more closely to patient prognoses [30], which is a relative objective and reliable measure. MGS-2 has been used to identify PPC development within abdominal surgery because of the high inter-rater reliability in the thoracic surgery population in recent years [22,24,31]. Accordingly, our present study screened PPCs based on the MGS-2 criteria. Our results showed that NAC inhalation reduced the incidence of PPCs, mechanical ventilation time, and ICU stay.

MGS-2 is a comprehensive evaluation system that translates and quantifies symptoms. While the broad criteria include a range of pulmonary complications that may contribute to pulmonary morbidity and mortality after liver transplantation [2,27,32]. Slight atelectasis, pneumothorax, or pleural effusion might be related to surgical manipulations and are usually self-limiting [27], thus having no obvious effect on the overall recovery of patients. The differences between the two methods might explain the different results in our study. On the one hand, NAC inhalation was shown to provide patients with good respiratory tract conditions, which supported early recovery and 12-month survival. On the other hand, our findings suggest that MGS-2 might be more reliable than the broad definition for diagnosing PPCs in patients who underwent OLT because it was more closely associated with patient prognoses. Further studies with larger samples are needed to confirm our results.

NAC has antioxidant and anti-inflammatory activities [12,33]. Because of the ability of increasing GSH concentrations in bronchoalveolar lavage fluid (BALF) and decreasing H_2_O_2_ concentration in EBC of patients with COPD, NAC administration showed favorable clinical outcomes of COPD [34]. NAC treatment could not only improve systemic oxygenation and reduce the need for ventilatory support in patients presenting with ALI but also have a protective effect on graft function in clinical liver transplantation, as confirmed by some experimental and clinical studies [35–37]. However, previous studies have administered NAC intravenously. Aerosol/inhalation therapy has been used as a supplementary means to treat patients with asthma, COPD, and cystic fibrosis for decades [35]. Compared with oral or intravenous administration, aerosol/inhalation medication is delivered by special devices via which that can rapidly and directly act on the airways to allow high local drug concentrations and limit systemic impact. Our study showed that preoperative and intraoperative NAC inhalation not only decreased the incidence of PPCs, but also reduced the 12-month mortality related to pulmonary infection. Inhaled NAC might give the good condition to respiratory tracts to benefit patients underwent OLT in regard to PPCs and outcomes which were confirmed in our present study.

In the current study, we found different lung infections including fungal infection, combined infection, and bacterial infection occurred after liver transplantation. The NAC group showed less fungal infections, combined infections and unknown infections, but significant more bacterial infection than control group. However, NAC has been found as an antibacterial drug by affecting bacterial biofilm formation [38]. This paradoxical phenomenon maybe due to the limitation of fungal and other unknown infections, resulting in the dysbacteriosis and dominant bacterial strains growth. In Yin et al. study [39], they also found intravenous administration of NAC increased biofilm formation by interaction with transferrin and subsequently elevated colonization of *Staphylococcus aureus* and *Pseudomonas aeruginosa* on implanted catheters, indicating that whether NAC could prevent bacterial infections may due to the infection status.

Some potential mechanisms were involved in the development of PPCs after OLT including the excessive oxidative stress and inflammatory responses [36,37]. Recently, many studies showed that biomarkers including TNF-α, IL-8, SP-D, CC16, and ICAM-1 were considered to be related to inflammatory responses and were demonstrated to be associated with ALI/ARDS. Markers in BALF were shown to be particularly sensitive [40,41]. EBC, a potential surrogate of BALF, contains many biomarkers that reflect the respiratory local inflammatory condition [42]. Our previous study demonstrated that TNF-α and IL-8 in EBC could be used as predictors of ARDS after OLT [24]. In accordance with the results in serum of patients, the present study found that NAC reduced the concentrations of TNF-α, IL-8, CC16, ICAM-1, and SP-D in EBC. These findings suggested that inhaling NAC could alleviate the inflammatory response and injury to the lungs, which might be an important mechanism to reduce the incidence of PPCs.

Our study has some limitations. First, it was a single-blinded, single-center study. Therefore, some bias might exist. Nevertheless, the results of our prospective randomized controlled clinical trial indicated significant advantages not only in the early postoperative period but also after 1 year of survival. These results may inspire multicenter studies with larger sample sizes. Second, the optimum NAC inhalation dose and administration time were not investigated. Third, a scanning electron microscopy study was not performed.

## Summary

In conclusion, our findings suggest that preoperative and intraoperative NAC inhalation may reduce the incidence of PPCs, which might be a good prospect to improve patient outcome after liver transplantation.

## Supporting information

**Supplementary Table 1:** The Melbourne Group Scale Version 2 (Criteria for Diagnosis of a Postoperative Pulmonary Complication)

**Supplemental Table 2:** Definitions of Postoperative Pulmonary Complications

## Abbreviations

ALI: acute lung injury
ARDS: acute respiratory distress syndrome
BALF: bronchoalveolar lavage fluid
CC16: Clara cell secretory protein
COPD: chronic obstructive pulmonary disease
EBC: exhaled breath condensate
ETCO_2_: end-tidal CO_2_
GSH: glutathione
I/R: ischemia–reperfusion
ICAM: intercellular adhesion molecule
IL: interleukin
MELD: model for end-stage liver disease
NAC: *N*-acetylcysteine
OLT: orthotopic liver transplantation
PPC: postoperative pulmonary complication
SOD: superoxide dismutase
SP-D: surfactant protein-D
TNF: tumor necrosis factor
