# Supplementary material for: N-Acetylcysteine inhalation improves pulmonary function in patients received liver transplantation
Source: Biosci Rep. 2018 Sep 28;38(5):BSR20180858. doi: 10.1042/BSR20180858 (PMC6165840; doi:10.1042/BSR20180858)
Supplement: Supplementary file 1 [file bsr20180858_Supp1.pdf]

Supplementary TABLE 1.

The Melbourne Group Scale Version 2 (Criteria for Diagnosis of a Postoperative Pulmonary Complication)

Diagnosis confirmed when four or more of the following are present:
• Chest radiograph report of consolidation/collapse
• Raised temperature > 38°C on two or more consecutive days
• Pulse oximetry saturation of oxygen (SpO_2) < 90% on room air on two consecutive days
• Production of yellow or green sputum which is different to preoperative assessment
• An otherwise unexplained white cell count > 11×10^9 L^{-1} or prescription of an antibiotic specific for respiratory infection
• Physician diagnosis of chest infection
• Presence of infection on sputum culture report
• Abnormal breath sounds on auscultation which differ from preoperative assessment

Supplementary TABLE 2.

Definitions of Postoperative Pulmonary Complications

Diagnosis confirmed by two independent doctors:

Acute lung injury

Acute onset of hypoxemia (partial pressure of oxygen in arterial blood [PaO_2]/fraction of inspired oxygen [FIO_2] ≤ 300 mmHg) with new bilateral infiltrates in the setting of either a normal pulmonary arterial wedge pressure (PAWP ≤ 18 mmHg) or the absence of suspected of left atrial hypertension when PAWP is not available.

Acute respiratory distress syndrome (ARDS)

ARDS is a special type of acute lung injury. In the setting of more severe hypoxemia (PaO_2/FIO_2 ≤ 200 mmHg), the term ARDS is applied.

Pleural effusion

Chest radiograph demonstrating blunting of the costophrenic angle, evidence of displacement of adjacent anatomical structures, or (in supine position) a hazy opacity in one hemithorax, with preserved vascular shadows.

Atelectasis

Collapse of the alveoli, lung opacification with shit of the mediastinum, hilum, or hemidiaphragm toward the affected area, and compensatory overinflation in the adjacent nonatelectatic lung.
